# Polyethylene Microplastics and Human Cells: A Critical Review

**DOI:** 10.3390/toxics13090756

**Published:** 2025-09-05

**Authors:** Sharin Valdivia, Camila Riquelme, María Constanza Carrasco, Paulina Weisser, Carolina Añazco, Andrés Alarcón, Sebastián Alarcón

**Affiliations:** 1 Cancer Biology Laboratory, Facultad de Medicina, Universidad San Sebastián, Sede Concepción, Campus Tres Pascualas, Concepción 4080871, Chile; sharin.valdivia@uss.cl (S.V.); alarcon.andres1707@gmail.com (A.A.); 2 Departamento de Ciencias Biológicas y Químicas, Facultad de Ciencias, Universidad San Sebastián, Sede Concepción, Campus Tres Pascualas, Concepción 4080871, Chile; 3 Escuela de Medicina, Facultad de Medicina, Universidad San Sebastián, Sede Concepción, Campus Tres Pascualas, Concepción 4080871, Chile; camila.riquelme@uss.cl (C.R.); constanza.carrasco@uss.cl (M.C.C.); paulina.weisser@uss.cl (P.W.); 4 Nutritional Biochemistry Laboratory, School of Nutrition and Dietetics, Faculty of Rehabilitation and Quality of Life Sciences, San Sebastian University, Valdivia 5091000, Chile; carolina.anazco@uss.cl

**Keywords:** microplastics, polyethylene, cellular effects

## Abstract

The widespread production and poor management of plastic waste have led to the pervasive presence of microplastics (MPs) in environmental and biological systems. Among various polymers, polyethylene (PE) is the most widely produced plastic globally, primarily due to its use in single-use packaging. Its persistence in ecosystems and resistance to degradation processes result in the continuous formation of PE-derived MPs. These particles have been detected in human biological matrices, including blood, lungs, placenta, and even the brain, raising increasing concerns about their bioavailability and potential health effects. Once internalized, PE MPs can interact with cellular membranes, induce oxidative stress, inflammation, and apoptosis, and interfere with epigenetic regulatory pathways. In vitro studies on epithelial, immune, and neuronal cells reveal concentration-dependent cytotoxicity, mitochondrial dysfunction, membrane disruption, and activation of pro-inflammatory cytokines. Moreover, recent findings suggest that PE MPs can induce epithelial-to-mesenchymal transition (EMT), senescence, and epigenetic dysregulation, including altered expression of miRNAs and DNA methyltransferases. These cellular changes highlight the potential role of MPs in disease development, especially in cardiovascular, metabolic, and possibly cancer-related conditions. Despite growing evidence, no standardized method currently exists for quantifying MPs in human samples, complicating comparisons across studies. Further, MPs can carry harmful additives and environmental contaminants such as bisphenols, phthalates, dioxins, and heavy metals, which enhance their toxicity. Global estimates indicate that humans ingest and inhale tens of thousands of MPs particles each year, yet long-term human research remains limited. Given these findings, it is crucial to expand research on PE MP toxicodynamics and to establish regulatory policies to reduce their release. Promoting alternative biodegradable materials and improved waste management practices will be vital in decreasing human exposure to MPs and minimizing potential health risks.

## 1. Introduction

Since the plastic industry began in the 1950s, global plastic production has grown rapidly, becoming essential to modern society. So far, over 8.3 billion tons of plastic have been produced, with estimates suggesting that annual production could reach 3.4 billion metric tons by 2050 [1]. Plastics are widely used due to their beneficial properties, including low production costs, durability, lightweight characteristics, and versatility across various industries such as packaging, construction, agriculture, medical devices, electronics, and textiles [2]. However, the same properties that make plastics valuable are also responsible for one of the greatest environmental challenges of our time: plastic pollution [3]. Plastics are synthetic organic polymers used to create a multitude of products. From a chemical perspective, they are biochemically inert polymers [4].

There are various types of plastics: polyethylene (PE), polypropylene (PP), polyvinyl Chloride (PVC), polyethylene terephthalate (PET), and polystyrene (PS) stand out [2]. Among these, studies show that PE is the most widely produced and used plastic, accounting for approximately 30% of the global plastic market [5]. PE is the most widely produced polymer worldwide, mainly used in the packaging industry for flexible films, bottles, and containers due to its chemical resistance and versatility. It is also heavily used in conduction systems and coatings in construction and agriculture, such as high-density pipes, geomembranes, and agricultural films. Additionally, its applications extend to biomedical and industrial fields, where it performs exceptionally well in prosthetics, medical devices, electrical insulators, and durable engineering parts [2,5]. Despite efforts to promote recycling and waste management, only a small percentage of plastic waste is effectively recycled—estimated at just 9% globally—while the majority ends up in landfills, is incinerated, or is released into the environment [6]. Unfortunately, plastics, including PE, persist in ecosystems. They undergo fragmentation through physical, chemical, and biological processes, resulting in the formation of MPs [7]. Since exposure is inevitable, it is crucial to understand their potential biological impacts.

**Figure 1:**
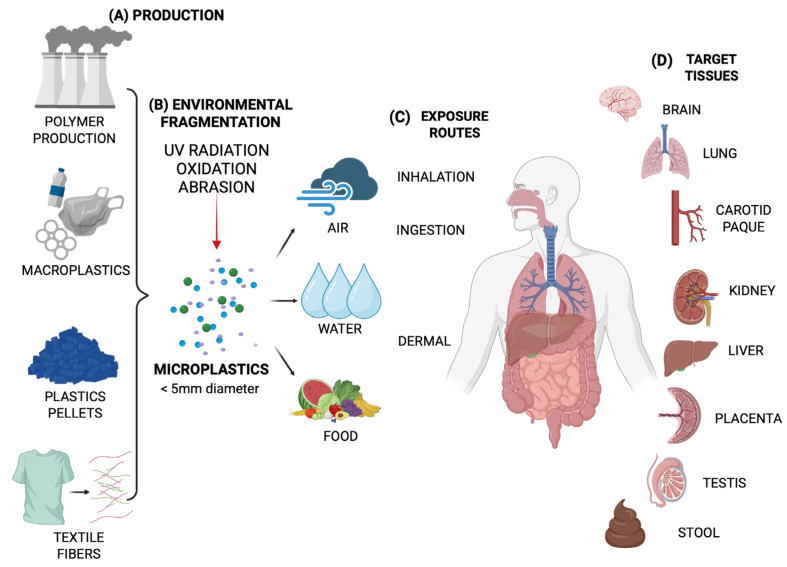
Life cycle of polyethylene and routes of human exposure to microplastics: (**A**) mass production and its primary use in single-use packaging; (**B**) environmental fragmentation caused by ultraviolet radiation, abrasion, and oxidation, which forms microplastics (MPs); (**C**) known pathways into the human body, including ingestion of food and water, inhalation of atmospheric dust, and skin contact; and (**D**) systemic distribution of MPs in various tissues and organs, such as the liver, lungs, placenta, and brain. Overall, this life cycle demonstrates the environmental persistence of PE and its possible effects on human health. Created in BioRender. Alarcón, S. (2025) (Accessed on 25 June 2025).

MPs are defined as synthetic polymer particles that have a maximum dimension between 1 micrometer (µm) and 5 mm (mm). The size range is divided into larger fractions (1–5 mm) and smaller fractions (1 µm to 1 mm). Nanoplastics (NPs), the smallest recognized fraction of plastic debris, are specifically defined as particles ranging in size from 1 nanometer (nm) to 1 micrometer (µm), thereby existing within the nano-scale [8,9,10]. Both types of particles have been found in air, soil, freshwater, marine environments, and even in biological tissues, including the human placenta, blood, and lungs [8,9,10]. This extensive distribution underscores their potential for bioaccumulation and human exposure (Figure 1). The degradation of PE results not only in physical particles but also in the release of a complex cocktail of chemical additives, including phthalates, bisphenols, flame retardants, and other persistent organic pollutants, known to exert endocrine-disrupting and cytotoxic effects [11,12]. 

Recent research suggests that MPs can interact with human cells in multiple ways, including disrupting membrane integrity, inducing oxidative stress, inflammation, apoptosis, and interfering with cellular transport mechanisms [13]. These findings underscore significant concerns regarding the long-term health implications of chronic MP exposure in humans. Although research on microplastics is available, the majority concentrates on polystyrene (PS) or environmental mixtures. PE, despite its widespread use, has been comparatively understudied in human cell models. This gap hampers the capacity to accurately evaluate genuine health risks. This review examines the production, degradation, and environmental fate of PE MPs, with a particular emphasis on their cellular and molecular effects. We critically examine recent in vitro and in vivo studies investigating how PE derived MPs interact with biological systems, their potential to contribute to disease development, and the urgent need for public health and regulatory responses.

## 2. Degradation of Polyethylene

**Figure 2:**
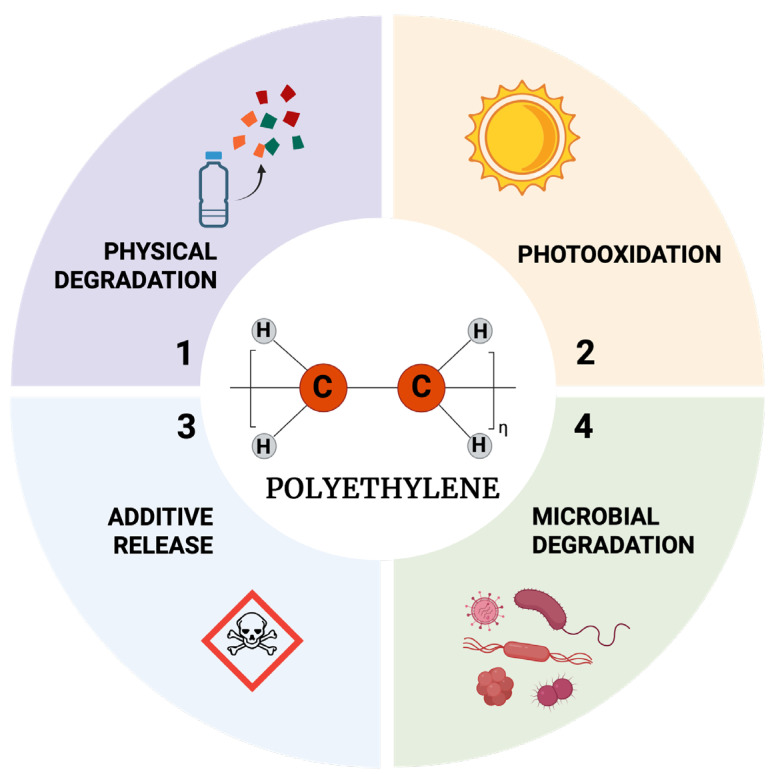
PE Degradation Pathways and Microplastic Formation. The diagram delineates the primary mechanisms through which PE degrades within the environment: (1) physical degradation via abrasion and mechanical fragmentation; (2) photodegradation and thermooxidation, wherein the formation of free radicals and polymer chain scission lead to the generation of microplastics and byproducts such as ketones, aldehydes, and carboxylic acids; (3) leaching of additives (including phthalates, flame retardants, bisphenols) and adsorption of environmental pollutants onto the aged polymer surface; and (4) microbial biodegradation, which proceeds through five stages: colonization and biofilm formation, biodeterioration, biofragmentation into monomers, assimilation, and mineralization to CO_2_/CH_4_ and biomass. Collectively, these processes contribute to the persistence of PE in ecosystems and to the formation of microplastics, which may have potential implications for human health. Created in BioRender. Alarcón, S. (2025) (Accessed on 25 June 2025).

All plastic polymers, including PE, can degrade through physical, chemical, and biological processes, resulting in the production of persistent environmental particles [14]. Because of its widespread use and resistance to degradation, PE is a major source of microplastics [15]. PE breaks down into small particles through physical, chemical, and biological processes. Environmental factors, including ultraviolet (UV) radiation, temperature, salinity, oxygen levels, and the presence of degrading microorganisms, significantly impact the rate and extent of PE degradation (Figure 2) [16].

Photodegradation is a primary factor causing polymer degradation in the environment. PE consists of backbone chains formed by C-C bonds, which do not hydrolyze and resist photodegradation due to the absence of UV-visible chromophores. However, impurities and structural defects acquired during manufacturing can act as chromophores [17,18]. During its degradation, PE forms free radicals, terminal vinyl groups, and ketone groups cleaved from the main chain. These free radicals react with ambient oxygen, producing peroxides. Through degradation, compounds such as alcohols, carboxylic acids, ketones, aldehydes, or esters may be generated, leading to the scission of the polymer chain, releasing MPs to the environment [17,19]. A high percentage of PE disposed of in open landfills degrades through photodegradation. However, this process is slow and inefficient, as UV light only affects the PE located on the surface [20].

Biodegradation is another method through which polymers can be broken down. However, because of its chemical characteristics, the biodegradation of PE is a slow and often incomplete process [21]. For example, the specific surface degradation rate (SSDR) of HDPE (High Density Polyethylene) ranges from 0 to ~11 μm per year in the marine environment.

Thus, the average lifespan of an HDPE bottle in an aquatic environment can reach 58 years [17]. In this context, several investigations have been conducted to find biological systems that can accelerate the degradation process of different polymers; Bacteria, fungi and yeasts capable of degrading PE have been reported [22]. PE biodegradation by microbes involves five stages: 1. Colonization, during which polysaccharides and biosurfactants are secreted to form a biofilm. This biofilm alters the chemical properties of the PE surface, making it more accessible to bacteria. 2. Biodeterioration, the oxidation process of carbonyls on the PE surface caused by oxygen, temperature, and UV radiation from sunlight. 3. Biofragmentation, where enzymes break down polymers into monomers that the microorganism can assimilate. 4. Assimilation, which occurs in the microorganism’s cytoplasm. This stage happens when microorganisms utilize the small biomolecules produced in the previous stage as sources of carbon and nitrogen to generate energy, biomass, and other metabolites. 5. Mineralization, which results in the production of biomass alongside the release of methane, CO_2_, and water [22,23].

The degradation of PE not only results in the formation of MPs, but also leads to the release of a wide array of chemical substances into the environment. These chemicals can be broadly categorized into two groups: (1) additives and residual monomers incorporated during the manufacturing process, and (2) environmental pollutants adsorbed onto the plastic surface post-production. Additives are intentionally introduced during polymer synthesis to enhance specific material properties such as durability, flexibility, coloration, and flame retardancy [12]. Among these, inert fillers are frequently used to improve mechanical strength and performance. Common examples include asbestos, glass fibers, rutile, silica, talc, various clays, chalk, aluminum oxide, soot, and carbon nanotubes [24]. Plasticizers, another widespread class of additives, are introduced between polymer chains to reduce intermolecular forces, thereby increasing the elasticity, softness, and resilience of the final material [25]. Stabilizers are added to enhance thermal and photochemical stability, protecting plastics from degradation due to ultraviolet (UV) radiation, heat, and oxidative conditions. Additional additive categories include dyes, lubricants, anti-adhesive agents, and flame retardants, all of which contribute to the functional versatility of plastic materials [24].

While it is true that additives are widely used throughout much of the production line, they are not chemically bonded to the plastic. Additives do confer many properties to plastics; however, many of these products are toxic [26]. Given that, as mentioned above, a high percentage of plastics are not handled properly after use and are released into the environment, the harmful effects on various species are unimaginable. The toxic effects of plastics on various aquatic organisms have been described. There is limited evidence regarding studies in humans, but several research groups around the world are focused on this topic [12,24,27]. Various substances considered dangerous by the European Union are found in many plastic products we use daily. These include bisphenol A (BPA), phthalates, and certain flame retardants [28]. Plastics can have harmful or toxic effects through two mechanisms: they can physically damage cellular structures in various organisms or release toxic substances contained within the plastic into the environment, such as Endocrine Disrupting Chemicals (EDC) and other harmful compounds [12]. EDCs are exogenous substances with hormonal activity that disrupt the homeostasis of the endocrine system. Several studies have demonstrated that these compounds can disrupt the function of multiple endocrine organs, including the hypothalamus, thyroid gland, testes, and ovaries [29]. In addition to the previously mentioned BPA [30] and phthalates [31], other concerning chemicals include alkylphenols [32], dioxins [27], and diphenyl ethers [33]. These EDCs act on different levels, such as hormone or membrane receptors, signaling pathways, secretion, transport and bioavailability [29,34,35,36,37]. Notably, these detrimental effects have been reported across different ages, including prenatal and childhood stages. For instance, phthalate exposure during pregnancy altered total T3 and FT4 plasma concentrations in pregnant women [38], and during childhood, phthalate exposure causes a notable reduction in thyroid gland weight and hyperactivity of this organ [39]. Various evidence shows that MPs transport toxic chemicals such as bisphenols, phthalates, dioxins, polybrominated diphenyl, aromatic hydrocarbons, heavy metals, and other substances added to the plastic during its manufacturing process [40]. For example, within EDCs, we find plasticizers such as phthalates. These EDCs are not covalently bonded to the plastic; thus, they can easily be released from the plastic and contaminate food and water, exposing us to toxic substances without our awareness [40]. These substances are known for their endocrine-disrupting effects, genotoxicity, immunotoxicity, and carcinogenic potential [11,12]. In addition to their intrinsic toxicity, MPs derived from PE can be associated with toxic chemicals present in the environment [41,42,43]. The list of concerning chemicals includes heavy metals [44], pathogenic microbes [45], and known carcinogenic compounds like polycyclic aromatic hydrocarbons (PAHs) [46] and polychlorinated biphenyls (PCBs) [47].

Therefore, exposure to MPs represents a significant health concern, as epithelial tissues may come into direct contact with toxic substances adsorbed onto their surfaces [48]. Moreover, the ability of both MPs to act as vectors for environmental contaminants substantially amplifies their overall toxicological impact. They carry harmful substances into the body, where they can accumulate and cause health issues. Consequently, degradation products of PE and other plastic polymers are not only intrinsically hazardous but also pose compounded risks due to their capacity to carry and deliver a wide range of external contaminants [49,50].

In summary, the PE degradation process involves complex mechanical, chemical, and biological transformations that ultimately generate MPs and NPs of this polymer. Additionally, these particles, along with the chemical compounds they may transport and deliver to the environment, pose a risk to both the ecosystem and human health. In this context, it is crucial to develop research aimed at understanding PE degradation processes in various environments and their effects on human health, while also designing effective mitigation strategies such as developing biodegradable alternatives and new recycling technologies.

## 3. Polyethylene and Byproducts: Cellular Interactions

PE MPs, once internalized, may elicit a wide range of systemic effects through their persistence, bioaccumulation, and ability to carry toxic chemicals. These MPs have been recovered from human blood [9], lung [10], human stool [51], semen [52], testicle [53], placenta [54], urine [55], hair [56], breast milk [57], sputum [58], meconium and infant faeces [59] suggesting that these particles may have a systemic distribution. Multiple studies using human cell models have reported various biological effects caused by treatment with PE MPs [13,60].

In this context, a study evaluated the cytotoxicity of MPs on the human cell lines Caco-2, HepG2, and HepaRG to detect any possible impact on the first organs that come into contact with ingested particles: the intestine and the liver. The results of the study demonstrate that particularly 1–4 μm PE MPs were transported to a small but significant extent through the intestinal epithelium in vitro, to a substantially greater degree than PS particles of the same size [61]. A study on T98G and HeLa cells exposed to 0.05 mg/L of PE (3–16 µm) showed differential effects. Under the experimental conditions described, exposure to PE MPs did not significantly alter cell viability in either of the tested cell lines. However, a selective increase in reactive oxygen species (ROS) production was observed in various types of human-origin cells (Table 1). ROS are bioproducts of cellular metabolism. They consist of a range of molecules with oxidizing properties known as ROS. Although these molecules are often associated negatively with aging and various diseases, their important role in cellular signaling is clear. ROS regulate several biological processes, including inflammation, proliferation, and cell death [62].

**Figure 3:**
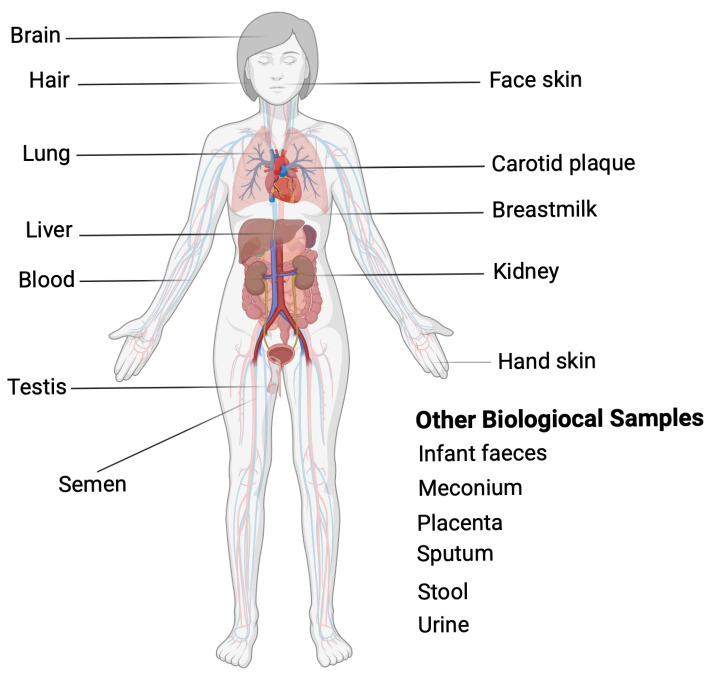
Evidence of PE in human tissues. The figure illustrates the detection of polyethylene (PE) microplastics within various human biological matrices. Particles have been identified in brain [63], hair [56], lung [10], liver [63], blood [9], testis [53], semen [52], face skin [56], carotid plaque [76], breastmilk [57], kidney [63], hand skin [56], infant faeces [59], meconium [59], placenta [54], sputum [58], stool [51] and urine [55], thereby confirming the capacity of these contaminants to traverse physiological barriers, accumulate within organs, and circulate systemically. The occurrence of PE in vital tissues, such as the brain and placenta, highlights the potential toxicological risks associated with chronic microplastic exposure to human health. Created in BioRender. Alarcón, S. (2025) (Accessed on 25 June 2025).

A study found PE MPs in human brain, kidney, and liver samples (Figure 3) [63]. Another recent study indicates that brains exhibited higher concentrations of MPs than liver or kidney samples. PE was the predominant polymer; the relative proportion of PE MPs was greater in brain samples than in liver or kidney [64]. These findings show the urgent need for more research to understand the pathways of exposure, absorption, and elimination of MPs, as well as the potential health consequences of MP exposure in living beings.

**Table 1:** Oxidative Stress and Inflammatory Mechanisms Induced by Polyethylene Microplastics/Nanoplastics in Human Cell Models.

Mechanism	Evidence	Model	Key findings	Reference
Oxidative Stress	Increase Mitochondrial Superoxide	Caco-2, HT-29	Decrease Viability	[65]
	Increase NO	Caco-2, A549	Decrease Viability	[66]
	Increase ROS	HaCaT	Down-regulation of cell growth and proliferation inhibition	[67]
	Increase ROS	T98G, Hela	Cell-type specific response	[68]
	Increase NO and ROS	THP-1, Jurkat, U937	Increase Viability	[66]
	Increase ROS, Activation TLR4/NOX2 axis	NP cells	Alteration cell morphology, Senescence cell	[69]
	Increase ROS	HCASMCs	Decreased Viability	[70]
Inflammation	Induce secretion of inflammatory cytokines IL-1β and IL-6	THP-1	Alteration of morphology and reduces cell density	[71]
	Increased transcripts for p53 and BMF, Increase LDH, Galectin-3 and RUNX-2, decreased α-SMA	HCASMCs	Induced apoptosis and altered migration and proliferation	[72]
	Increased TNF-α, IL-6, Caspase-1 and VOCs	HCASMCs	Decreased Viability	[70]
	Lysosomal Dysfunction (induced autophagosome formation and increased p62 expression	THP-1	Decreased Viability	[73]
	Phagocytosis of PE	MDM	Increased Survival	[74]

To date, no longitudinal epidemiological studies have been conducted in humans to assess the potential health effects of MP exposure. Nevertheless, multiple studies using animal models and human cell lines have demonstrated that chronic exposure to MPs is associated with metabolic dysregulation, immune system modulation, and an increased susceptibility to inflammatory and fibrotic pathologies. Emerging in vitro data suggest that even MPs cross the blood–brain barrier and alter neuronal homeostasis. Interestingly, an article shows increased accumulation of MPs in a cohort of human deceased brains diagnosed with dementia, with notable deposition in cerebrovascular walls and immune cells [63]. Recently, a study suggested that MPs can infiltrate human bone, cartilage, and intervertebral discs through the bloodstream, resulting in specific patterns of MPs accumulation; this invasion may affect skeletal health by altering the expression of inflammatory and bone morphogenetic cytokines [75]. Similarly, MPs are emerging as a potential risk factor for cardiovascular disease in preclinical studies. A study found that 58% of patients had PE in their carotid artery plaque, and 31 of those patients (12.1%) also had a measurable amount of polyvinyl chloride in the carotid plaque [76]. Similarly, a research group found MPs in the blood samples of 34 out of 36 participants. They specifically determined that 39% of the samples contained PE MPs. It is important to note that the analyzed samples did not feature a single type of plastic. This study indicates that the number of MP particles in blood was higher among individuals with advanced education levels and that the percentage of plastic containers among total vessels in the refrigerator was significant.

Alarmingly, the authors of this study suggest that higher MP particle concentrations in blood were linked to altered coagulability proteins [77]. Another study shows the presence of various types of MPs, including PE, in human lung tissue extracted from patients who had surgery for lung issues cancer [10]. On the other hand, the presence of PE MPs has also been noted in 50% of lung samples taken from autopsies of individuals living in Sao Paulo [78].

Emerging evidence indicates that MPs, particularly those derived from PE, can disrupt multiple biological systems by interfering with cellular processes. Critical determinants such as particle size, shape, surface charge, and the ability to adsorb and transport environmental contaminants enhance their interaction with human cells and contribute to a wide range of pathophysiological effects [79]. Regardless of the route of exposure, the plasma membrane constitutes the first cellular barrier encountered by MPs, mediating their potential biological impact [80]. In vitro studies using HepG2 cells have demonstrated that PE MPs compromise membrane integrity in a concentration-dependent manner. These particles adhere to the cell surface and can insert into the lipid bilayer, forming pores that cause structural damage to dipalmitoylphosphatidylcholine (DPPC), a major phospholipid component of cell membranes [80]. Similarly, other reports have shown that PE particles induce lipid membrane destabilization through mechanical stretching [81], compromising the membrane’s barrier function and potentially allowing the influx of additional harmful agents. On the other hand, a study of carotid artery plaque extracted from patients in whom the presence of PE MPs was detected shows an increase in interleukin-18, interleukin-1β, tumor necrosis factor α (TNF-α), interleukin-6, and an abundance of collagen, CD3, and CD68 in relation to plaques where the presence of MPs was not detected [76].

Studies in human cell lines show that exposure to PE MPs induces the epithelial–mesenchymal transition (EMT). Traversa et al. show that acute treatment with PE MPs alter cell morphology but does not impact cell proliferation and viability in human bronchial (BEAS-2B) and alveolar (A549) epithelial cells. They demonstrate that exposure to PE MPs induces modulation of EMT markers, such as Snail 1, Snail 2, Zeb1, Vimentin, N-cadherin, and E-cadherin, in the BEAS-2B and A549 cell lines. Interestingly, the effects of PE MP exposure on cell morphology and EMT induction can be reversed after the removal of PE MPs [82]. In human vaginal keratinocytes, exposure to PE MPs induces a sustained alteration in the expression of DNA methyltransferase and DNA demethylase, which may impact epigenetic regulatory processes, leading to accelerated cell aging and inflammation, or an increased risk of malignant transformation [83]. The study above also describes the deregulation of various miR expressions (miR-29a, miR-34a, miR-124a, miR-125b, miR-145, miR-200c, and miR-378a) after exposition to PE MPs [83]. Currently, the largest body of evidence regarding the ability of PE MPs to modulate the epigenome is found in animal models [84,85,86]. Here, dose-related effects of PE MPs on hematological, genotoxic, and epigenetic aspects in mammals have been described, highlighting the potentially hazardous health effects of environmental PE MPs [86].

All this data indicates that chronic exposure to PE MPs can affect various cellular processes and may ultimately lead to different clinical manifestations that impact an individual’s health. Recently, a study found MPs in the tissues of patients diagnosed with colorectal cancer (CRC). These studies show that, compared to adjacent non-cancerous tissues, tumor tissues exhibited a greater variety and distribution of MPs, mainly PE and PVC [87].

Similarly, a study found MPs in 14 of 18 human follicular fluid samples [88]. So far, animal studies have shown the negative effects of MPs on ovarian function. The impact of MPs in human follicular fluid and reproductive health remains unknown.

## 4. Limitations of Current Evidence

Although there is growing evidence that PE MPs are present in human tissues and may be toxic, there are still major limitations in detecting, measuring, and regulating them. These challenges hinder the development of comprehensive public health policies and impede the creation of guidelines based on robust evidence for exposure.

Identifying and characterizing MPs in biological samples like blood, lung tissue, placenta, or brain tissue poses distinct analytical challenges. Right now, there is no one-size-fits-all standard method for isolating and analyzing MPs (Table 2). Polymer type and particle morphology are frequently determined using techniques such as Raman Microscopy and FTIR. However, these methods are restricted when applied to small particles, nanoplastics (less than 1 µm), and complex biological samples due to interference from organic material autofluorescence [89,90]. Emerging methods like Pyrolysis-Gas Chromatography–Mass Spectrometry (Py-GC/MS) and Thermal Extraction Desorption-GC/MS (TED-GC/MS) provide greater sensitivity and accuracy for identifying plastic types but are destructive and lack spatial resolution [89]. Asymmetric Flow Field-Flow Fractionation (AF4) coupled with Multi-Angle Light Scattering (MALS) and Inductively Coupled Plasma Mass Spectrometry (ICP-MS) have shown promise for nanoparticle characterization, although accessibility and standardization remain limited [91]. Another major obstacle is sample contamination during collection and processing. This can occur due to plastic lab materials, airborne MP particles, and cross-contamination from consumables, which can lead to false positives. To prevent this, strict contamination control and validation with procedural blanks are essential, but these practices are not always applied consistently across studies [92].

Despite all the available evidence, it is important to understand the higher in vitro doses are often chosen to observe mechanistic effects within short experimental periods and to simulate potential effects of long-term cumulative exposure or localized tissue deposition [12,93,94]. Therefore, further studies are necessary to evaluate the potential effects of MP PE on humans. It is important to note that most mechanistic insights related to oxidative stress, inflammation, membrane disruption, and epigenetic changes are derived from in vitro experiments with human cell lines, often conducted at concentrations much higher than those found in human tissues [13]. These models are invaluable for revealing potential toxicological pathways, but their findings should be viewed as hypotheses about possible biological responses rather than definitive evidence of human health effects. In contrast, detecting polyethylene microplastics in human biological samples (blood, placenta, lung, brain, etc.) provides real-world evidence of bioaccumulation [13]. However, the levels identified are consistently lower than those used in experiments, and the clinical effects remain mostly unknown. For this reason, we have clearly distinguished mechanistic observations from cell models and the implications suggested by human biomonitoring studies, highlighting the urgent need for long-term clinical research to fill this knowledge gap [79,95,96].

Although detecting PE MPs in human samples is a significant advance, the current evidence is limited by several methodological problems. Most studies have small sample sizes and use different analytical methods, often with varying size-detection thresholds and sensitivity to nanoplastics [97]. Additionally, the lack of standardized protocols for sample collection and processing increases the risk of airborne or laboratory contamination, which can result in false positives [89,97]. Conversely, as previously delineated, certain analytical methodologies compromise the integrity of the samples, while others are constrained by the detection limits and may present potential interferents within the matrix embedding the sample. All of these factors are critical considerations in the quantification and detection of MPs [89,91,98]. As a result, comparing results across studies is difficult, and the clinical significance of the MP concentrations in different types of human samples remains unclear [13,79]. Future research should focus on standardizing pre-analytical procedures, implementing strict contamination controls, and integrating complementary analytical platforms to improve both sensitivity and accuracy [89]. Large-scale, long-term studies that incorporate clinical biomonitoring and follow-up are vital for establishing causal links between exposure to PE MPs and their potential impacts on human health [95,96].

**Table 2:** Comparative Performance of Analytical Techniques for Detecting and Characterizing Microplastics/Nanoplastics in Biological Matrices.

Technique	Minimun Detectable Size	Strengths	Limitations	References
µFT-IR ^(1)^	≥20 μm	Fast, extensive polymer library. Non-destructive technique	Matrix interference (biofilms or organic matter) can affect spectral quality. Limited sensitivity to fine particles	[89,99]
µ-Raman ^(2)^	≈1 μm	Typifies sub 10 μm particles. Non-destructive technique	Difficult analysis of embedded or coated particles. Heat-sensitive polymers can be damaged by powerful lasers	[89,99]
Py-GC/MS ^(3)^	Determine mass	Highly sensitive mass quantification	Requires polymer standards for quantification. It analyzes the total mass of polymers, without discriminating between individual particles	[98,99]
TED-GC/MS ^(4)^	Determine mass	Rapid polymer screening	It does not provide morphological information. It does not discriminate between individual particles	[99]
AF4-MALS ^(5)^	~10–20 nm	Separate by size, quantify mass	Does not identify polymer type. Not applicable to solid samples. Sensitive to aggregation.	[91]
AF4-MALS-ICP-MS ^(6)^	~10–20 nm	Characterizes nano-metal/plastic complexes	It does not identify the polymer type. It does not provide morphological information.	[91]

µFT-IR ^(1)^: Fourier Transform Infrared Microspectroscopy; µ-Raman ^(2)^: Raman Microspectroscopy; Py-GC/MS ^(3)^: Pyrolysis coupled to Gas Chromatography and Mass Spectrometry; TED-GC/MS ^(4)^: Thermal Extraction Desorption coupled to Gas Chromatography and Mass Spectrometry; AF4-MALS ^(5)^: Asymmetric Flow Field-Flow Fractionation coupled to Multi Angle Light Scattering; AF4-MALS-ICP-MS ^(6)^: Asymmetric Flow Field-Flow Fractionation Multi Angle Light Scattering Inductively Coupled Plasma Mass Spectrometry.

## 5. Regulatory Landscape and Policy Challenges

Currently, there is no universally accepted standard for MP levels in food, water, or living tissues. Unlike chemical pollutants, MPs are not yet part of the routine monitoring programs at most environmental and public health agencies. Recently, the European Union has taken regulatory action by adding MPs to the REACH Regulation ((EC) No 1907/2006). This move bans the intentional use of MPs in cosmetics, detergents, and some industrial applications [100]. Similarly, the U.S. Microbead-Free Waters Act of 2015 bans plastic microbeads in rinse-off cosmetics, but it does not cover secondary MPs, such as those from PE fragmentation in the environment [101]. Additionally, the development of biodegradable materials, more efficient recycling systems, and public policies aimed at reducing the production of single-use plastics is necessary [102].

National policies also differ greatly. Some countries have enacted partial bans on plastic bags or single-use items, but few have addressed MPs in food or biological exposure. Chile, for example, passed Law No. 21.368 (2021) banning single-use plastics in commercial establishments, yet it does not include regulations on PE MPs or monitoring of human exposure [103]. In 2019, the Ministry of Food and Drug Safety of Korea developed a testing method for detecting microbeads in cosmetic products. Subsequently, in 2021, strict regulations were enacted concerning household chemical products such as fabric softeners, bleach, deodorant, and laundry detergent, specifically regarding the permissible levels of MPs they contain. In the United Kingdom, the prohibition on the manufacture and sale of microbead-containing personal care products was established in 2021. Furthermore, in 2022, India implemented a ban on single-use plastics with low utility and high littering potential [104]. To date, international organizations such as the World Health Organization (WHO), in its 2022 report, acknowledge the presence of MPs in drinking water, but concluded that the available data are insufficient to draw firm conclusions about the risks to human health, highlighting the need for further research and better detection methods [95].

## 6. Future Prospects

As described during the development of this article, the presence of PE MPs and other types of plastics has been noted in various human tissues, indicating that contamination from these particles has transcended the environmental sphere to become a global health concern problem [96,105]. Evidence shows that regardless of the origin of PMs, they are transported in water and by wind [106]. After inhalation or ingestion, in animal models, MPs have been described as translocating from organs of the digestive and respiratory systems to the circulatory system, resulting in an inflammatory response [107,108,109].

Studies have found MPs in different environments such as beaches, seawater, and seafloor sediments [110]. These toxic particles have also been found ubiquitously in various types of foods for human consumption such as seafood [111], salt and honey [112,113], and milk [114], among others [115,116]. Global estimates place the annual ingestion of MPs between 39,000 and 52,000 particles, rising to 74,000–121,000 when inhalation is included [117]. On the other hand, research published in 2023 determined that adult Korean people ingested through food about 1.4 × 10^−4^ to 3.1 × 10^−4^ g of MPs per week [118]. Similarly, a group investigated the presence of MPs in tap water from various regions around the world, discovering MPs in 87% of the 1148 samples collected. Notably, most of the MPs detected were smaller than 50 µm [119]. On the other hand, a study of tap water from various locations in Spain indicates that the concentration of MPs in water is extremely low, far below the ng/L range. The results from this group suggest that MPs in drinking water do not represent a significant route of exposure to MPs and likely pose a low risk to human health [92]. As of now, there are no standardized methodologies or protocols for quantifying MPs detected in various biological matrices, making it challenging to compare results obtained by different research groups. This necessitates harmonized MPs extraction protocols and broader investigations into regional and individual variations in MP exposure [120].

Given the growing number of routes involved in MPs’ human exposure, this is a concern for the potential impact related to the development of diseases. Despite being exposed to MPs through various sources, the implications for human health still remain in a nebulous area of knowledge. Multiple studies have detected the presence of PP, PET, PS and PE in samples or biological fluids (Figure 3) [96,105]. For example, a study on semen samples collected from men in Southern Italy shows the presence of MPs (PE, PP, PS, PVC, and PET, among others) in six of the ten samples [52]. Another study involving 17 samples (11 livers, 3 spleens, and 3 kidneys) indicated that the livers of patients with liver cirrhosis showed elevated concentrations of MPs (PS, PVC, PET) compared to liver samples obtained from individuals without liver disease. To date, whether the accumulation of MPs in cirrhotic liver samples is a cause or a consequence of this pathology remains to be determined [121]. Recently, a group from the University of Bergen, Norway indicated that MPs were detected in 17 out of 18 fecal samples, with PP, PE, and PS being the predominant MPs. This shows that there was no significant association between MPs abundance and seafood consumption, or with other food groups, such as chocolate and chips. These researchers suggest that dietary habits alone may not be the primary determinant of MP exposure [120]. In contrast, a group of researchers from Lanzhou University in Lanzhou, China, studied the feces of 122 volunteers and found that frequent consumption of take-out food may increase the intake of MPs, thereby altering the gut microbiota and metabolites of young adults, and could pose a potential risk factor for obesity [122].

Nevertheless, there exists literature that contradicts the aforementioned descriptions. Numerous studies found that MPs made from different polymers had no significant effects on cell lines of various origins and mouse models [60]. Within this context, certain studies have indicated an absence of cellular effects following exposure to PE MPs. For instance, a study utilizing an intestinal cell model demonstrated that healthy and inflamed cells exhibit differing susceptibilities to PE particles sized 0.2 μm to 9 μm (NPs and MPs), with exposure of inflamed cultures to 50 mg cm^−2^ of PE failing to elevate the levels of the inflammatory cytokine IL-8, in contrast to healthy cultures [123]. Furthermore, neither healthy nor inflamed cultures showed a significant increase in pro-inflammatory cytokines, including IL-1β, IL-6, and TNF-α, nor did they exhibit DNA damage at a concentration of 50 mg cm^−2^ PE MPs [123]. Consistent with these findings, another research group reported no substantial fluctuations in lactate dehydrogenase levels in Caco-2 cells exposed to PE particles ranging from 30 to 140 μm for 48 h at a concentration of 1000 mg L^−1^ [124]. The information above indicates that, under certain experimental conditions—especially with larger particles and in specific cell types or states—PE MPs may not cause noticeable cytotoxic, inflammatory, or genotoxic effects. This implies that their toxicity heavily depends on the context and particular physicochemical properties. However, the lack of immediate effects does not rule out potential long-term risks or risks in different exposure scenarios.

Overall, tackling the challenge of MP pollution will require a multidisciplinary approach that combines molecular biology, environmental medicine, public health, nanotechnology, and regulatory policies. The focus should extend beyond merely understanding the potential toxicity of these compounds; it must also include preventing exposure and reducing their entry into the food chain.

Given the global scope of plastic pollution and growing evidence of human exposure, we need urgent and coordinated action worldwide. This should involve: (1) developing reliable and standardized methods to detect MPs in human samples; (2) establishing biomonitoring programs for at-risk populations; (3) creating interdisciplinary frameworks that combine environmental science, molecular biology, and public health; and (4) setting toxicological reference doses and exposure limits for high-risk plastics like PE. Until such regulations are in place, precautionary measures—such as cutting single-use plastic consumption, improving waste management, and investing in biodegradable alternatives—remain the most effective public health strategies.

## 7. Conclusions

PE MPs have emerged as more than an environmental concern—they represent a growing threat to human health. Their presence in multiple human biological matrices, including blood, lungs, placenta, and even brain tissue, underscores their capacity to penetrate physiological barriers and accumulate systemically. However, the concentrations reported are consistently lower than those used in experimental models, and their clinical consequences remain largely undefined.

Experimental evidence demonstrates that PE MPs can disrupt cellular homeostasis by damaging membranes, increasing oxidative stress, triggering inflammation, and altering epigenetic regulation. These effects vary depending on particle size, surface properties, and their ability to carry toxic environmental contaminants.

Although definitive causal links in humans remain unconfirmed due to the absence of longitudinal clinical studies, the convergence of in vitro and in vivo findings supports the need for precautionary measures. Regulatory actions targeting plastic production, usage, and waste management are urgently needed to mitigate exposure risks.

Overall, PE MPs should be recognized as emerging pollutants of high concern. Addressing their impact requires not only scientific investigation but also systemic changes in how plastics are produced, consumed, and regulated in modern society. Finally, there is a limited understanding of the bioaccumulation potential, biodistribution, and elimination mechanisms of MPs in the human body. Existing reports of MP detection in blood, placenta, and organs are based on small sample sizes and often lack rigorous contamination control. Taken together, these limitations underscore the urgent need for standardized methodologies, longitudinal human studies, and interdisciplinary research to better characterize the toxicodynamics and health risks associated with environmental exposure to PE MPs.

## Data Availability

No new data were created or analyzed in this study. Data sharing is not applicable to this article.

## Abbreviations

The following abbreviations are used in this manuscript:

PE	Polyethylene
PP	Polypropylene
PVC	Polyvinyl Chloride
PET	Polyethylene Terephthalate
PS	Polystyrene
MPs	Microplastics
SSDR	Specific Surface Degradation Rate
HDPE	High Density Polyethylene
EDC	Endocrine Disrupting Chemicals
PAHs	Polycyclic Aromatics Hydrocarbons
ROS	Reactive Oxygen Species
EMT	Epithelial–Mesenchymal Transition
